# Professional identity and engagement in oral health promotion activities among oral health ambassadors in China: a national cross-sectional survey

**DOI:** 10.3389/fpubh.2026.1881408

**Published:** 2026-08-21

**Authors:** Jiakun Fang, Hongzhao Yu, Shuai Hao, Wensheng Rong

**Affiliations:** 1 Office of Operations Management, Peking University School and Hospital of Stomatology & National Center of Stomatology & National Clinical Research Center for Oral Diseases & National Engineering Research Center of Oral Biomaterials and Digital Medical Devices, Beijing, China; 2 China Oral Health Foundation, Beijing, China; 3 China Medical Tribune, Beijing, China; 4 Department of Preventive Dentistry, Peking University School and Hospital of Stomatology & National Center of Stomatology & National Clinical Research Center for Oral Diseases & National Engineering Research Center of Oral Biomaterials and Digital Medical Devices, Beijing, China

**Keywords:** China, health promotion, oral health, professional identity, public health communication, workforce engagement

## Abstract

**Background:**

Sustained engagement of frontline health communication professionals is essential for effective public health promotion. While traditional material incentives show limited efficacy in maintaining intrinsic motivation, identity-based designation programs have emerged as a potential alternative, yet their impact in large-scale, real-world public health settings remains underexamined.

**Methods:**

A cross-sectional survey was conducted among all registered members of the China Oral Health Foundation Oral Health Promotion Ambassador program in June 2025. An anonymous online questionnaire was distributed via the program's official digital platform, yielding 1,071 valid responses from all 31 provincial-level regions of China. The questionnaire assessed sociodemographic characteristics, health communication practices, self-perceived impact of the ambassador designation, and practice-related challenges and needs. Self-reported significant enhancement of professional identity was the primary outcome. Chi-square tests were used for univariable screening, and binary logistic regression was employed to identify factors independently associated with significant self-reported identity enhancement.

**Results:**

The majority of respondents (96.4%) reported that the ambassador designation enhanced their professional identity, with 57.1% reporting significant enhancement. In multivariable analysis, nursing staff showed a significantly higher likelihood of significant identity enhancement compared to junior/attending-level dentists (OR = 1.595, 95% CI: 1.103–2.306, *p* = 0.013). Annual participation frequency was independently associated with significant enhancement, with both the 6–10 times/year group (OR = 1.490, 95% CI: 1.104–2.012, *p* = 0.008) and the >20 times/year group (OR = 1.832, 95% CI: 1.232–2.725, *p* = 0.003) showing significantly higher likelihood compared to the lowest-frequency group. Ambassadors in Western China showed a significantly lower likelihood of significant enhancement relative to Eastern China (OR = 0.710, 95% CI: 0.514–0.980, *p* = 0.037). Officially recognized certificates (83.4%) were the most preferred incentive, ranked above material rewards (41.8%).

**Conclusions:**

Following the ambassador designation, participants reported improvements in professional identity, and enhancement was significantly associated with engagement frequency and professional roles. However, the heterogeneity in responses across regions and roles suggests that uniform empowerment is insufficient. Public health managers should transition toward stratified incentive architectures and provide targeted resources that address the specific structural challenges faced by different practitioner subgroups.

## Introduction

The effective delivery of population-level health promotion and education depends heavily on the sustained engagement of personnel involved in public health communication (1, 2). However, frontline health communication practitioners worldwide often face problems such as heavy workloads, limited organizational support and a lack of formal recognition for their non-clinical contributions (3, 4). These challenges create a critical barrier to maintaining their motivation and continuous participation in health communication activities (2, 4, 5). Although traditional incentive structures in healthcare have often centered on financial rewards, autonomous forms of motivation have been shown to more consistently predict employee performance (6). This has generated growing interest in non-material mechanisms that strengthen occupational identity and professional belonging as critical pathways to sustained engagement (7).

Theories such as social identity theory (SIT) and self-determination theory (SDT) provide a theoretical basis for understanding how occupational identity and professional belonging sustain workforce engagement. SIT proposes that individuals derive part of their self-concept (the set of attributes and roles one uses to define oneself) from valued group memberships, such that a stronger professional group identity motivates behaviors consistent with that group's collective mission (8). SDT emphasizes that fulfilling psychological needs for competence, autonomy and relatedness enhances autonomous motivation, performance and wellbeing (1, 9). Together, these theories suggest that formal recognition mechanisms which strengthen group membership and fulfill core psychological needs hold potential for reinforcing professional identity and sustaining engagement.

Despite their theoretical relevance, motivational correlates of formal professional identity designation remain underexamined in real-world public health systems, especially within large-scale programs. The oral health field represents a particularly relevant context for this inquiry, given that health communication here faces distinctive structural challenges rooted in both audience behavior and professional culture. Oral diseases remain among the most prevalent chronic conditions globally, yet preventive behaviors are consistently underadopted (10, 11). In China, oral health literacy remains low and care-seeking patterns prioritize treatment over prevention (11), posing potential challenges for frontline communicators (such as dentists, dental hygienists, and oral health nurses) who have historically been positioned within a professional culture emphasizing clinical over preventive roles (12, 13). These conditions make the question of what sustains communicator motivation particularly salient in the oral health context. One response to these challenges has been the development of structured public health programs that formally designate healthcare professionals as oral health communicators. The national Oral Health Promotion Ambassador program, established by the China Oral Health Foundation (COHF) in 2019, provides a unique context to examine the associations between formal identity designation, professional identity enhancement, and engagement patterns within a large-scale, real-world public health program. As of June 2026, the program comprised 22,365 certified members across all 31 provincial-level regions of China and reached an estimated 24.85 million audience members (14).

The present study examines this national program, with three aims: (1) to characterize the health communication practices of ambassador program members; (2) to examine self-reported changes in professional identity and practice behaviors following ambassador designation; (3) and to identify factors independently associated with self-reported significant professional identity enhancement, with particular attention to the role of practitioner characteristics and engagement intensity. The research aims to provide evidence-based insights for public health managers and policymakers, helping them design effective and sustainable strategies to motivate the health communication workforce. Although grounded in the Chinese oral health context, the research addresses challenges that are broadly relevant to health systems worldwide in sustaining workforce engagement in preventive and public health services.

## Materials and methods

### Survey design and participants

A cross-sectional survey among all registered members of the COHF Oral Health Promotion Ambassador program. An anonymous online questionnaire was distributed to all registered program members through the COHF official digital platform in June 2025. Although all registered program members were invited to participate, participation was voluntary, resulting in a convenience sample rather than a true census. The study was conducted in accordance with the Declaration of Helsinki. It was reviewed by the Ethics Committee of Peking University School and Hospital of Stomatology, which determined that formal ethical approval was not required because the study only involved a non-interventional, anonymous survey of healthcare professionals. Electronic informed consent was obtained from all participants before questionnaire completion. All participants provided informed consent prior to completion. A total of 1,071 valid responses were obtained (response rate of 8%), with representation from all 31 provinces consistent with the overall geographical distribution of the program membership. Because participation required both self-enrollment in the program and voluntary completion of the survey, participants may represent a more motivated subgroup within the cohort. A CHERRIES checklist is provided in Supplementary Table 1. For the purposes of this study, the ambassador designation was conceptualized as a form of identity empowerment: a formal mechanism through which an authoritative national body confers professional recognition upon frontline health communicators. Drawing on SIT, membership in the certified ambassador cohort was expected to reinforce occupational worth and group belonging, motivating behaviors aligned with the program's collective mission (8). Professional identity enhancement was treated as the primary measurable outcome of this empowerment process, assessed through participants' self-reported perceptions of change following designation. A structured questionnaire was developed based on literature review and expert consultation to ensure the validity and understandability of the content. Six experts from fields such as national health policies, oral preventive medicine, health promotion and popularization and news dissemination reviewed the questionnaire. Following three rounds of expert review and item revision, the finalized 31-item questionnaire was evaluated for content validity using a four-point relevance scale (1 = not relevant, 4 = highly relevant). All 31 items achieved an item-level content validity index (I-CVI) of 1.00, indicating excellent content validity. The pilot test was conducted among a small sample of oral health ambassadors (*n* = 21) to evaluate item clarity and survey flow. Based on their feedback, minor wording adjustments were made to reduce ambiguity. The final questionnaire consisted of four sections (English translation available in Supplementary Table 2): (1) sociodemographic and professional characteristics (items 1–6), which collected data on participants' duration as an ambassador, geographic location, workplace type, professional role, educational level and years of clinical experience; (2) public health engagement practices (items 7–11), which assessed participation frequency, target audiences and preferred communication formats; (3) impact of the ambassador title (items 12–15), which used retrospective questions to measure self-perceived changes, with professional identity enhancement as primary outcome (item 15); and (4) perceived challenges and needs (items 16–31), assessing difficulties, resource shortages and incentive preferences.

Several measures were implemented to ensure data quality. All questionnaire items were mandatory, resolving the issue of missing data. Each survey link was restricted to a unique IP address to prevent duplicate submissions. A minimum response time of 1 min was required for submission to ensure sufficient reading and response time. Logical consistency checks were performed to identify any contradictory responses by manual confirmation. An electronic informed consent statement was displayed on the first page of the survey, and submission of the questionnaire indicated consent to participate.

To assess the internal structure of the perceived challenge items, an exploratory factor analysis was conducted on the four core difficulty items (insufficient time, lack of high-quality materials, low patient acceptance, and insufficient policy support), confirming a single-factor solution (KMO = 0.793, Bartlett's *p* < 0.001) that explained 71.58% of the total variance, with acceptable internal consistency (Cronbach's α = 0.862).

### Statistical analysis

All statistical analyses were performed using SPSS version 26.0 (IBM Corp., Armonk, NY, USA). Descriptive statistics were used to characterize the sample and current practices. For the regression analysis, the professional identity enhancement was transformed into a binary variable. The responses of “significantly enhanced” were classified as the significant enhancement group and all others were categorized as the comparison group. Chi-square tests were used for univariable analysis to examine associations between each candidate variable and significant professional identity enhancement. For the annual participation frequency variable, which has a natural ordinal ordering, a trend chi-square test was additionally applied to examine the presence of a linear association. A binary logistic regression model was then constructed, incorporating sociodemographic, professional, and behavioral variables simultaneously to identify independent predictors of significant professional identity enhancement. ORs and their 95% confidence intervals (CIs) were calculated. A *p*-value of < 0.05 was considered statistically significant.

## Results

### Sample characteristics and univariable analysis

The sociodemographic and professional characteristics of the sample are presented in Table 1. The sample comprised primarily junior/attending-level dentists (42.8%) and nursing staff (17.9%) from public hospitals and private dental institutions, with a high level of popularization activity (over half of members participated six or more times annually). Univariable analysis identified geographical region (χ^2^ = 10.817, *p* = 0.013), workplace type (χ^2^ = 6.020, *p* = 0.049), professional role (χ^2^ = 10.211, *p* = 0.037), and annual participation frequency (χ^2^ = 9.201, *p* = 0.027) as significantly associated with significant professional identity enhancement. Duration as ambassador, educational level, and years of clinical experience were not significantly associated (*p* > 0.05). Trend chi-square analysis further confirmed a statistically significant positive linear trend between annual participation frequency and the proportion reporting significant identity enhancement (*p* = 0.008).

**Table 1:** Sample characteristics and univariable analysis of significant professional identity enhancement (*N* = 1,071).

Variable	Category	Total (*N* = 1,071) *n* (%)	Significant enhancement (*N* = 611) *n* (%)	Other (*N* = 460) *n* (%)	χ^2^	*p*-value
Duration as ambassador					0.261	0.877
	1–2 years	624 (58.3)	352 (56.4)	272 (43.6)		
	3–4 years	232 (21.6)	135 (58.2)	97 (41.8)		
	≥5 years	215 (20.1)	124 (57.7)	91 (42.3)		
Geographical region					10.817	**0.013**
	Eastern China	501 (46.8)	289 (57.7)	212 (42.3)		
	Central China	204 (19.0)	120 (58.8)	84 (41.2)		
	Western China	243 (22.7)	120 (49.4)	123 (50.6)		
	Northeastern China	123 (11.5)	82 (66.7)	41 (33.3)		
Workplace type					6.020	**0.049**
	Public hospital	445 (41.5)	235 (52.8)	210 (47.2)		
	Private dental institution	495 (46.2)	294 (59.4)	201 (40.6)		
	Primary care clinic & other	131 (12.3)	82 (62.6)	49 (37.4)		
Professional role					10.211	**0.037**
	Junior/attending-level dentist^a^	458 (42.8)	255 (55.7)	203 (44.3)		
	Senior-level dentist^b^	167 (15.6)	81 (48.5)	86 (51.5)		
	Nursing staff	192 (17.9)	123 (64.1)	69 (35.9)		
	Other oral health professional^c^	110 (10.3)	64 (58.2)	46 (41.8)		
	Other^d^	144 (13.4)	88 (61.1)	56 (38.9)		
Educational level					2.062	0.357
	College diploma	313 (29.2)	186 (59.4)	127 (40.6)		
	Bachelor's degree	578 (54.0)	330 (57.1)	248 (42.9)		
	Master's degree or above	180 (16.8)	95 (52.8)	85 (47.2)		
Years of clinical experience					1.746	0.418
	< 5 years	266 (24.8)	161 (60.5)	105 (39.5)		
	5–15 years	451 (42.1)	252 (55.9)	199 (44.1)		
	>15 years	354 (33.1)	198 (55.9)	156 (44.1)		
Annual participation frequency					9.201	**0.027**
	1–5 times	468 (43.7)	245 (52.4)	223 (47.6)		
	6–10 times	314 (29.3)	189 (60.2)	125 (39.8)		
	11–20 times	123 (11.5)	70 (56.9)	53 (43.1)		
	>20 times	166 (15.5)	107 (64.5)	59 (35.5)		

^a^Includes resident and attending dentists according to the National Professional Title System for Healthcare Professionals in China. ^b^Includes associate chief and chief dentists. ^c^Includes dental technicians, oral public health professionals, and oral health researchers. ^d^Includes general public health educators, administrative staff, students, and other personnel who participated in oral health promotion activities. Bold values indicate *p*-value < 0.05.

### Health promotion practices

The main target audiences for oral health promotion were children (89.4%), adolescents (73.8%), middle-aged and elderly adults (67.3%), young parents (57.3%), pregnant women (9.3%), and people with disabilities (2.9%), with participants permitted to select up to three options. All practice-related items were assessed using ranking scales on which lower mean rank values indicate greater frequency of use or higher perceived ease of learning. Regarding communication formats, free dental clinics were used most frequently (mean rank 1.751 ± 0.24), followed by community lectures (3.131 ± 0.39), one-on-one counseling (3.551 ± 0.71), short-form videos (3.671 ± 0.38), infographic or text-based posts (3.771 ± 0.33), and live-streaming (5.121 ± 0.23). In terms of teaching content, scientific tooth-brushing technique was addressed most frequently (mean rank 1.100 ± 0.37), followed by dental floss use (2.090 ± 0.45), interdental brush use (3.280 ± 0.59), and mouthwash use (3.470 ± 0.76). For perceived audience learning difficulty, interdental brush use was rated most challenging (3.250 ± 0.83), followed by dental floss use (2.490 ± 0.81) and mouthwash use (2.201 ± 0.23), while scientific tooth-brushing technique was perceived as the easiest to convey (1.971 ± 0.11). A mismatch was observed between teaching prioritization and instructional challenge, as dental floss use ranked second in teaching frequency yet third in ease of learning, suggesting that content prioritization does not always align with what audiences find most accessible.

### Changes in practice following ambassador designation

The self-perceived impact of the ambassador title designation is detailed in Table 2. A majority of participants reported a positive impact on their professional identity, with 96.4% stating it was either significantly enhanced (57.1%) or somewhat enhanced (39.3%). Similarly, most respondents reported positive behavioral changes after becoming an ambassador, including an increased frequency of promotion activities (87.3%) and the use of more diverse communication formats (86.4%).

**Table 2:** Self-perceived impact of the ambassador title designation (*n* = 1,071).

Item	Category	*n*	%
Impact on professional identity	Significantly enhanced	611	57.1
	Somewhat enhanced	421	39.3
	No effect	39	3.6
Changes in health promotion behavior (multiple-choice)^a^	Increased frequency of promotion activities	935	87.3
	More diverse promotion formats	925	86.4
	Greater focus on audience follow-up and feedback	677	63.2
	Other	38	3.6
Increased emphasis on basic skills teaching	Yes, a significant increase	918	85.7
	A slight increase	139	13.0
	No change	14	1.3
Greatest change in teaching specific skills	Scientific and effective brushing	746	69.7
	Dental floss usage	186	17.4
	Interdental brush usage	117	10.9
	Mouthwash usage	22	2.1

^a^“Changes in Health Promotion Behavior” was a multiple-choice question; percentages represent the proportion of respondents who selected each option, and the sum may exceed 100%.

### Predictors of significant professional identity enhancement

The binary logistic regression model was globally significant (Omnibus χ^2^ = 40.138, *p* = 0.002). Multicollinearity diagnostics confirmed model stability, with all VIF values below 5 and a maximum condition index of 16.219. Results are presented in Table 3.

**Table 3:** Binary logistic regression analysis of factors associated with significant professional identity enhancement (*n* = 1,071).

Variable	β	SE	χ^2^	*p*-value	OR	95% CI
Annual frequency of health promotion activities (ref: 1–5 times)			11.941	**0.008**		
6–10 times	0.399	0.153	6.774	**0.008**	1.490	(1.104, 2.012)
11–20 times	0.305	0.213	2.039	0.153	1.356	(0.893, 2.061)
>20 times	0.605	0.203	8.937	**0.003**	1.832	(1.232, 2.725)
Duration as ambassador (ref: 1–2 years)			1.078	0.583		
3–4 years	0.105	0.165	0.403	0.526	1.111	(0.803, 1.535)
≥5 years	0.175	0.178	0.964	0.326	1.191	(0.840, 1.690)
Geographical region (ref: Eastern China)			11.723	**0.008**		
Northeastern China	0.445	0.222	4.029	0.045	1.560	(1.011, 2.409)
Western China	−0.343	0.165	4.341	**0.037**	0.710	(0.514, 0.980)
Central China	0.109	0.181	0.358	0.549	1.115	(0.781, 1.591)
Workplace type (ref: public hospital)			2.711	0.258		
Primary care/other	0.341	0.230	2.210	0.137	1.407	(0.897, 2.207)
Private dental institution	0.197	0.160	1.512	0.219	1.218	(0.890, 1.667)
Professional role (ref: junior/attending-level dentist)			11.367	**0.023**		
Senior-level dentist	−0.303	0.215	1.985	0.159	0.738	(0.484, 1.126)
Nursing staff	0.467	0.188	6.150	**0.013**	1.595	(1.103, 2.306)
Other oral health professional	−0.167	0.239	0.489	0.485	0.846	(0.529, 1.352)
Other	0.115	0.223	0.250	0.617	1.122	(0.715, 1.760)
Educational level (ref: college diploma)			0.024	0.988		
Bachelor's degree	0.025	0.173	0.022	0.883	1.026	(0.730, 1.441)
Master's degree or above	0.012	0.244	0.003	0.959	1.013	(0.628, 1.634)
Years of clinical experience (ref: < 5 years)			0.739	0.691		
5–15 years	−0.160	0.187	0.735	0.391	0.852	(0.591, 1.229)
>15 years	−0.115	0.214	0.288	0.592	0.892	(0.586, 1.356)

Bold values indicate *p*-value < 0.05.

Regarding professional role, nursing staff demonstrated a significantly higher likelihood of significant identity enhancement compared to junior/attending-level dentists (OR = 1.595, 95% CI: 1.103–2.306, *p* = 0.013). No significant differences were observed for senior-level dentists or other professional categories. Geographical region was a significant predictor (χ^2^ = 11.723, *p* = 0.008). Using Eastern China as the reference, ambassadors in Northeastern China showed a significantly higher likelihood of significant identity enhancement (OR = 1.560, 95% CI: 1.011–2.409, *p* = 0.045), while those in Western China showed a significantly lower likelihood (OR = 0.710, 95% CI: 0.514–0.980, *p* = 0.037). Central China did not differ significantly from Eastern China. Regarding annual participation frequency, compared to the low-frequency group (1–5 times/year), both the 6–10 times group (OR = 1.490, 95% CI: 1.104–2.012, *p* = 0.008) and the >20 times group (OR = 1.832, 95% CI: 1.232–2.725, *p* = 0.003) showed significantly higher likelihood of significant enhancement. However, the 11–20 times group showed a positive but non-significant association (OR = 1.356, 95% CI: 0.893–2.061, *p* = 0.153). Workplace type, duration as ambassador, educational level, and years of clinical experience were not independent predictors of significant professional identity enhancement (*p* > 0.05).

### Heterogeneity in perceived challenges

**Figure 1:**
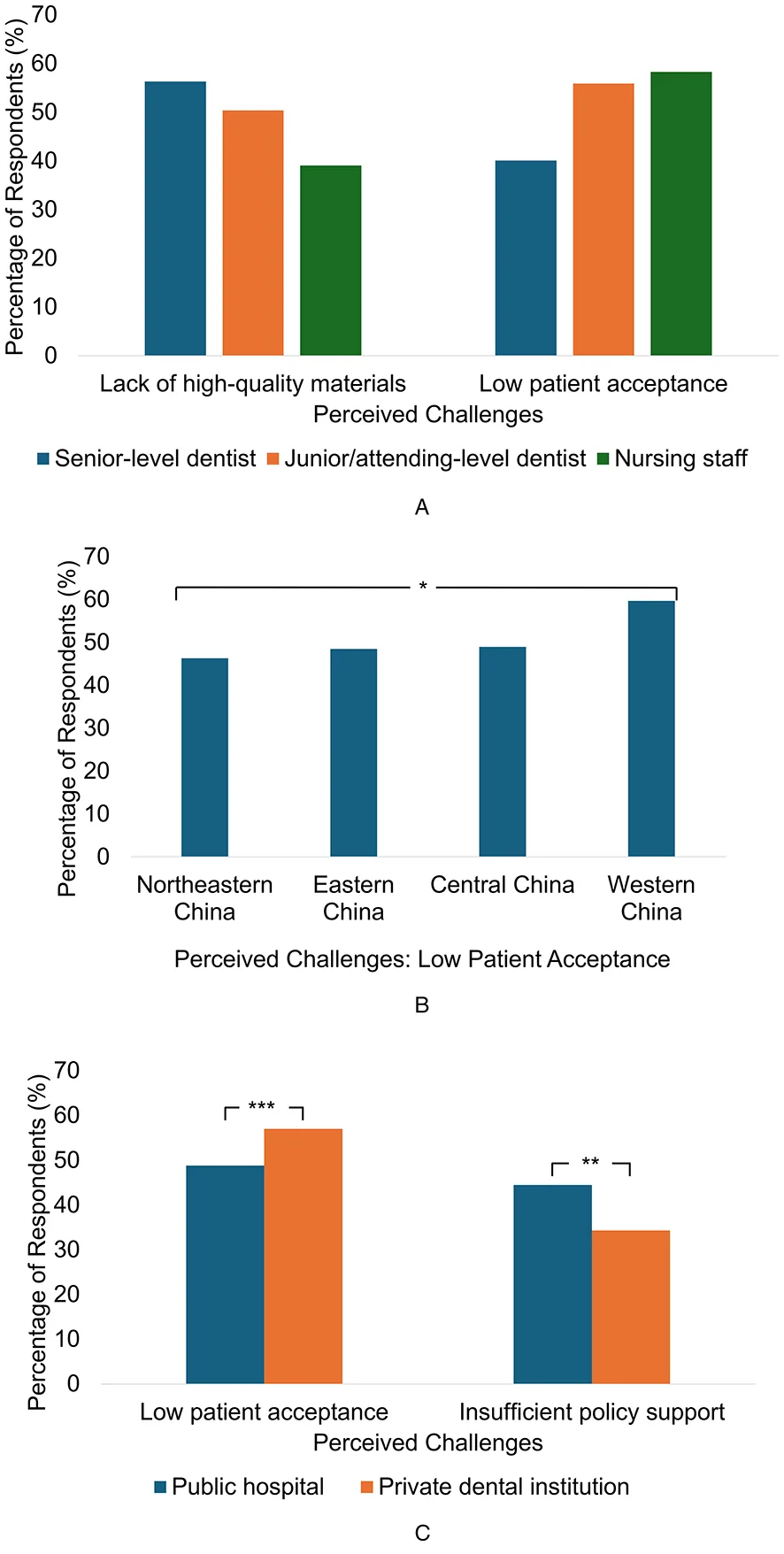
Heterogeneity in perceived challenges across subgroups. The figure displays the percentage of respondents reporting challenges, compared across **(A)** professional role, **(B)** geographical region and **(C)** workplace type. *p*-values are derived from Chi-square tests. *p < 0.05, **p < 0.01, ***p < 0.001.

Significant differences were found in the practical challenges and needs reported by participants (Figure 1). As can be seen from Figure 1A, senior-level dentists were most concerned about the lack of high-quality materials (56.3%), a proportion significantly higher than that of junior/attending-level dentists (50.4%) and nursing staff (39.1%), with χ^2^ = 18.123 and *p* = 0.001. In contrast, and nursing staff (58.3%) and junior/attending-level dentists (55.9%) perceived low patient acceptance as a more pressing issue compared to senior-level dentists (40.1%), with χ^2^ = 26.185 and *p* < 0.001. A significant geographical difference was observed regarding patient acceptance (χ^2^ = 9.943, *p* = 0.019). Ambassadors working in the western region of China reported this as a challenge more frequently (59.7%) compared to those in other regions (Figure 1B). By workplace type (Figure 1C), participants from private institutions were significantly more likely to report low patient acceptance as a major challenge (57.0%) compared to those in public hospitals (48.8%), with χ^2^ = 25.241 and *p* < 0.001. On the other hand, participants in public hospitals reported insufficient policy support more frequently (44.5%) than participants in private institutions (34.3%), with χ^2^ = 13.405 and *p* = 0.004.

### Implementation burdens, evaluation practices, and incentive preferences

The study also identified structural issues related to the implementation and evaluation of health promotion activities (Table 4). Regarding the largest time cost in practice, the most frequently mentioned by participants was follow-up and effectiveness evaluation (47.8%), rather than content preparation (32.7%) or the actual execution of activities (18.4%). This finding corresponds to the self-reported assessment method. Although many participants reported typically using informal feedback to confirm the impact of health communication on the audience, such as patient improvement at follow-up visits (76.8%), nearly one-third of respondents (30.8%) indicated that they currently lack effective evaluation methods.

**Table 4:** Perceived time costs, evaluation methods and incentive preferences (*n* = 1,071).

Item	*n*	%
Largest perceived time cost		
Follow-up and effectiveness evaluation	512	47.8
Content preparation (e.g., creating presentations, videos)	350	32.7
Actual execution (e.g., lectures, one-on-one guidance)	197	18.4
Other	12	1.1
Methods for evaluating effectiveness^a^		
Improvement in patients' oral health at follow-up visits	822	76.8
Audience feedback on behavioral changes (e.g., starting to use floss)	794	74.1
Assessing knowledge retention via questionnaires or tests	405	37.8
Currently lack of effective evaluation methods	330	30.8
Other	18	1.7
Desired incentives for health promotion work^a^		
Official certificates of recognition	893	83.4
Continuing education credits	683	63.8
Positive feedback from patients	666	62.2
Media publicity	628	58.6
Material rewards	448	41.8
Other	38	3.6

^a^“Methods for evaluating effectiveness” and “Desired incentives for health promotion work” were multiple-choice questions; percentages represent the proportion of respondents who selected each option, and the sum may exceed 100%.

In terms of expected incentives, the ambassadors considered non-material recognition related to professional values to be particularly important. 83.4% of participants indicated that officially recognized certificates were the most frequently requested incentive. This was followed by other non-material rewards such as continuing education credits (63.8%) and positive feedback from patients (62.2%). Material rewards (41.8%) were relatively ranked lower.

## Discussion

This study examined oral health communication practices, self-reported behavioral changes, and perceived challenges among members of a national identity designation program, and investigated associations between practitioner characteristics, engagement frequency, and the likelihood of significant professional identity enhancement.

### Identity empowerment and differential professional responses

Consistent with SIT, the Ambassador designation appears to serve as a formal professional identity anchor, repositioning participants as recognized oral health advocates within their professional field (8). This repositioning may fulfill core psychological needs identified by SDT: competence derived from health promotion practice, relatedness gained through membership in a goal-oriented professional community, and autonomy practiced through discretionary engagement choices (1). The fulfillment of these needs may theoretically explain the internalization of externally granted recognition into autonomous motivation, a pathway consistent with the self-reported attitudinal and behavioral changes observed in this study. This finding aligns with growing evidence that non-material recognition plays a vital role in motivating health professionals in public-facing roles (2, 4). The association between formal designation and professional identity enhancement is consistent with the broader literature on professional identity formation in health professional populations (15–17).

An important finding is the differential effect by professional role. Nursing staff showed a significantly higher likelihood of significant identity enhancement compared to junior and attending-level dentists (OR = 1.595, 95% CI: 1.103–2.306, *P* = 0.013). In traditional oral healthcare settings, nursing staff typically occupy auxiliary roles (18, 19) with limited independent access to professional recognition. The ambassador designation offers these practitioners a platform to show professional value, bypassing conventional hierarchical structures. Intrinsic and identity-based rewards produce stronger motivational responses precisely in professional groups where extrinsic recognition is structurally limited (20), suggesting that identity designation programs targeting practitioners with limited existing recognition pathways may yield stronger motivational returns.

### Participation frequency and its association with professional identity enhancement

Self-Perception Theory holds that individuals infer their internal attitudes and commitments by observing their own behaviors (21), suggesting that sustained participation in oral health promotion activities may serve as ongoing behavioral evidence of professional dedication, reinforcing the identity internalization initiated by the ambassador designation. In line with this account, annual participation frequency was a significant behavioral predictor of significant identity enhancement. Both the 6–10 times/year group (OR = 1.490, 95% CI: 1.104–2.012, *p* = 0.008) and the >20 times/year group (OR = 1.832, 95% CI: 1.232–2.725, *p* = 0.003) showed significantly higher likelihood of significant enhancement compared to the lowest-frequency group, with a statistically significant positive linear trend confirmed by trend chi-square analysis (*p* = 0.008). The 11–20 times/year group showed a directionally consistent OR of 1.356 (95% CI: 0.893–2.061, *p* = 0.153), suggesting that the positive association is present across the full participation range. The lack of statistical significance in this intermediate frequency band may reflect limited statistical power resulting from the relatively small subgroup size (*n* = 123), rather than a true plateau in the association. These findings align with prior evidence linking sustained engagement to occupational identity formation (15), pointing to active participation as a potentially reinforcing mechanism for professional identity among ambassador program members.

### Behavioral patterns following ambassador designation

The practical significance of professional identity enhancement lies in its potential association with observable changes in health promotion behavior. The present findings indicate that ambassador designation was accompanied by widespread self-reported changes in both professional identity and practice behaviors among program members. Following designation, 87.3% of respondents reported an increased frequency of promotion activities, 86.4% reported adopting more diverse communication formats, and 85.7% reported a significant increase in emphasis on basic oral health skills teaching. The convergence of these behavioral shifts across multiple dimensions of practice, including frequency, format diversity, and content emphasis, suggests that the ambassador designation is associated not merely with attitudinal change, but with a broader reorientation of professional practice toward health communication, consistent with theoretical accounts linking professional identity to occupational behavior as outlined by both SIT and SDT (1, 8).

While behavioral changes were assessed through retrospective self-report rather than objective measurement, the consistency of these patterns across diverse professional roles and institutional settings supports the interpretation that formal identity designation is meaningfully associated with practice-level engagement, adding to the limited empirical evidence on non-material recognition programs in large-scale health communication contexts (2, 4).

### Toward a stratified incentive architecture

Although formal identity designation appears associated with enhanced professional identity and engagement motivation, a gap between this activated motivation and the organizational support needed to sustain it is evident in the data. This gap was observed even within the highly motivated ambassador cohort, suggesting that structural barriers may be more pronounced in the broader health promotion workforce. Three structural imbalances are identified. The first is a front-end resource deficit. Across professional subgroups, a substantial proportion of ambassadors reported lacking access to high-quality promotional materials, ranging from 39.1% among nursing staff to 56.3% among senior-level dentists, suggesting that content resource gaps are pervasive regardless of professional background. The second is a midstream assessment gap. Nearly half of participants (47.8%) identified follow-up and effectiveness evaluation as their largest time burden, yet standardized evaluation tools remain absent for most practitioners. Without objective performance metrics, the program cannot distinguish high-impact from low-impact engagement, and practitioners receive insufficient feedback to improve their practice. The third is a back-end incentive misalignment. The strong preference for officially recognized certificates (83.4%) over material rewards (41.8%) confirms that participants seek symbolic acknowledgment of professional contribution rather than financial compensation. This preference for symbolic over material recognition suggests that participants value acknowledgment of their professional contributions and public health mission, a pattern consistent with public service motivation theory (22) and with evidence that recognition and professional development opportunities are central to sustaining engagement in health workforces (23).

These findings point toward a stratified incentive architecture aligned with practitioner engagement stages. The fact that these structural gaps were identified even among respondents who showed sufficient commitment to the program to voluntarily complete this survey further suggests that they reflect underlying program-level needs. For early-stage practitioners, priority should be given to reducing structural entry barriers through provision of ready-to-use communication resources, training in multimedia formats, and access to peer support networks. For practitioners with established engagement, evaluation frameworks should shift from activity volume toward composite indicators capturing audience reach, content quality, and behavioral change outcomes. For the most active practitioners, career development pathways such as peer mentor or program trainer designations can address higher-order professional development needs while multiplying program reach through a tiered practitioner community. Regional differentiation should also be considered. Geographical region was a significant predictor of professional identity enhancement (χ^2^ = 11.723, *p* = 0.008), and the regional variation observed may partly reflect disparities in oral health workforce density and institutional capacity across Chinese regions (24). Geographically targeted support strategies addressing both resource constraints and audience engagement barriers are therefore needed in underserved regions.

From a broader public health perspective, maintaining the motivation of large-scale frontline health promotion workforces remains a persistent challenge for health systems worldwide. Our findings suggest that formal professional identity recognition represents a promising non-material component of workforce engagement strategies. Rather than replacing conventional incentives, identity-based recognition may complement existing approaches by activating internalized motivation and reinforcing professionals' sense of purpose and public health contribution. The structural logic underlying the stratified incentive architecture may have potential applicability across preventive public health settings beyond oral health.

### Limitations

This study draws on a large-scale national program sample spanning all 31 provincial-level regions of China, offering an empirical window into identity-based incentive mechanisms operating at population scale. Nevertheless, several limitations should be noted. First, the single-group retrospective cross-sectional design precludes causal inferences. The high rate of self-reported professional identity enhancement should be interpreted as characterizing participants' subjective experiences rather than definitive causal effects of the program, as selection bias, expectancy bias, and potential reverse causality cannot be completely ruled out. Second, the 7.9% response rate and use of convenience sampling indicate the analytical sample represents a highly engaged subset, which may limit the generalizability of the findings to the broader health workforce. Furthermore, the core outcome variable of this study was measured using a retrospective, single-item self-assessment. This measurement approach may introduce measurement error due to recall bias and faces the potential risk of common method bias, which to some extent limits the accurate estimation of the association strength between professional identity and practice behaviors. Finally, grounded specifically in the operational context of oral health, these findings warrant further investigation to verify their applicability to other clinical specialties.

## Conclusions

Identity-based formal recognition, as exemplified by the national ambassador program, demonstrates meaningful positive associations with professional identity enhancement and increased health promotion engagement among frontline oral health communicators. These findings contribute to the growing evidence base on non-material recognition programs in public health workforce management and suggest that formal identity designation warrants further investigation as a potential approach to sustaining engagement within public health systems. Public health managers and policymakers should move beyond simply launching such identity programs and establish a supportive ecosystem that provides adequate resources, standardized evaluation tools and incentive structures that reward high-quality and sustainable engagement.

## Data Availability

The raw data supporting the conclusions of this article will be made available by the authors, without undue reservation.
