# Supplementary material for: Professional identity and engagement in oral health promotion activities among oral health ambassadors in China: a national cross-sectional survey
Source: Front Public Health. 2026 Aug 21;14:1881408. doi: 10.3389/fpubh.2026.1881408 (PMC13541924; doi:10.3389/fpubh.2026.1881408)
Supplement: Supplementary file 1 [file Table_1.docx]

| Supplementary Table 1. Checklist for Reporting Results of Internet E-Surveys (CHERRIES)^1^ | | |
| --- | --- | --- |
| **Item Category** | **Checklist Item** | **Explanation** |
| **Design** | Describe survey design | This was a cross-sectional study using an online survey. The target population was the national cohort of Oral Health Promotion Ambassadors in China. The sample is a convenience sample of this cohort, consisting of members who voluntarily responded to the survey invitation. |
| **IRB (Institutional Review Board) approval and informed consent process** | IRB approval | The study was conducted following the Declaration of Helsinki. According to the Ethics Committee of the Peking University School of Stomatology, no formal ethical approval was required, as the study involved a non-interventional, anonymous survey of healthcare professionals. Participants were informed about the purpose and procedure of the study and provided informed consent before participation. |
|  | Informed consent | The first page of the online survey served as an information sheet, detailing the study’s purpose, data anonymity, the investigators’ affiliations, and an estimate of the time required for completion (approx. 30 minutes). |
|  | Data protection | The survey was anonymous, and no personally identifiable information was collected. All data were stored on a secure, password-protected server accessible only to the core research team. |
| **Development and pre-testing** | Development and testing | The questionnaire was developed based on a comprehensive literature review and expert consultation with specialists in public health and dentistry. The usability and technical functionality of the online questionnaire were pre-tested by a small group of ambassadors to ensure clarity of questions and smooth navigation before its official launch. |
| **Recruitment process and description of the sample having access to the questionnaire** | Open survey versus closed survey | A closed survey. |
|  | Contact mode | Online. |
|  | Advertising the survey | The survey was announced and the link was disseminated via the official digital communication channels of the COHF, which are exclusively accessible to the program members. |
| **Survey administration** | Web/E-mail | A web-based platform. |
|  | Context | The survey link was distributed by COHF through its official communication channels used to contact all ambassadors. |
|  | Mandatory/voluntary | Voluntary |
|  | Incentives | No. |
|  | Time/Date | June 2025. |
|  | Randomization of items or questionnaires | Questionnaires. |
|  | Adaptive questioning | No. |
|  | Number of Items | 5 |
|  | Number of screens (pages) | 7 |
|  | Completeness check | Yes, all questions were set as mandatory fields on the survey platform. |
|  | Review step | Participants were able to move back and forth between pages to review and change their answers before final submission. |
| **Response rates** | Unique site visitor | Yes. IP address. |
|  | View rate (Ratio of unique survey visitors/unique site visitors) | N/A. |
|  | Participation rate (Ratio of unique visitors who agreed to participate/unique first survey page visitors) | 7.9%(1071/13557). |
|  | Completion rate (Ratio of users who finished the survey/users who agreed to participate) | 100%. All 1071 submitted questionnaires were fully completed. |
| **Preventing multiple entries from the same individual** | Cookies used | No. |
|  | IP check | Yes. |
|  | Log file analysis | No. |
|  | Registration | No. |
| **Analysis** | Handling of incomplete questionnaires | Not applied. Questionnaires were completed due to the mandatory field setting. |
|  | Questionnaires submitted with an atypical timestamp | No. |
|  | Statistical correction | Not applied. The goal was to analyze the characteristics of the responding cohort rather than to generalize to the entire population. |
| ^1^Eysenbach G. Improving the quality of Web surveys: the Checklist for Reporting Results of Internet E-Surveys (CHERRIES). J Med Internet Res. 2004 Sep 29;6(3):e34. doi: 10.2196/jmir.6.3.e34. | | |
