# Supplementary material for: Professional identity and engagement in oral health promotion activities among oral health ambassadors in China: a national cross-sectional survey
Source: Front Public Health. 2026 Aug 21;14:1881408. doi: 10.3389/fpubh.2026.1881408 (PMC13541924; doi:10.3389/fpubh.2026.1881408)
Supplement: Supplementary file 2 [file Table_2.docx]

| Supplementary Table 2. English translation of the questionnaire used in the study | | |  |
| --- | --- | --- | --- |
| **Item No.** | **Original Question (Chinese)** | **English Translation** | **Response Options (Format)** |
| **Part 1: Sociodemographic and Professional Characteristics** | | | |
| Q1 | 您成为健康口腔推广大使多长时间？ | How long have you been an Oral Health Promotion Ambassador? | 1 year; 2 years; 3 years; 4 years; 5 years or more (Single-choice) |
| Q2 | 您所在的省份是？ | In which province are you located? | Province (Single-choice) |
| Q3 | 您的工作单位类型是? | What is your type of workplace? | Public hospital; Private dental institution; Primary care clinic/Community health center; Other (Single-choice) |
| Q4 | 您的职称/身份是? | What is your professional title/role? | Junior/attending-level dentist; Senior-level dentist; Nurse; Other oral health professional; Other (Single-choice) |
| Q5 | 您的学历是? | What is your highest educational level? | College diploma; Bachelor’s degree; Master’s degree or above (Single-choice) |
| Q6 | 您的参与临床工作的年限是? | How many years have you been engaged in clinical work? | < 5 years; 5-10 years; 10-15 years; 15-20 years; > 20 years (Single-choice) |
| **Part 2: Public Health Promotion Practices** | | | |
| Q7 | 您平均每年参与口腔科普活动的次数是? | How many times per year do you participate in oral health promotion activities? | 1-5 times; 6-10 times; 11-20 times; > 20 times (Single-choice) |
| Q8 | 您的科普内容主要面向哪三类人群? | What are the top three target audiences for your health promotion content? | Children; Adolescents; Young parents; Middle-aged and elderly adults; Pregnant women; People with disabilities (Multiple-choice, select up to three) |
| Q9 | 以下几种口腔科普形式，您最常使用的是? | Please rank the following health promotion formats in order of frequency of use. | (1=Most frequent → 6=Least frequent) Free dental clinics; Community lectures; One-on-one guidance; Short-form videos; Posters; Live-streaming (Ranking) |
| Q10 | 请您对自身工作中以下四项技能的科普频次进行排序 | Please rank the frequency of teaching the following four skills in your work. | (1=Most frequent → 4=Least frequent) Scientific and effective brushing techniques; Dental floss usage; Interdental brush usage; Mouthwash usage (Ranking) |
| Q11 | 请对受众学会四项技能的难易程度排序 | Please rank the difficulty for the audience to learn the following four skills. | (1=Easiest to learn → 4=Hardest to learn) Scientific and effective brushing techniques; Mouthwash usage; Dental floss usage; Interdental brush usage (Ranking) |
| **Part 3: Impact of the Ambassador Title** | | | |
| Q12 | 成为大使后，您的科普行为是否有以下变化? | After becoming an Ambassador, have you observed the following changes in your health promotion behavior? | Increased frequency of promotion activities; More diverse promotion formats; Greater focus on audience follow-up and feedback; Other (please specify) (Multiple-choice) |
| Q13 | 成为大使后，您在科普内容上是否更侧重基本口腔保健技能的教学? | After becoming an Ambassador, do you place a greater emphasis on teaching basic oral hygiene skills (e.g., brushing, flossing)? | A significant increase; A slight increase; No change (Single-choice) |
| Q14 | 成为大使后，下面哪一项口腔保健技能您的教学变化最大? | After becoming an Ambassador, which of the following oral hygiene skills has seen the greatest change in your teaching? | Scientific and effective brushing; Dental floss usage; Interdental brush usage; Mouthwash usage (Single-choice) |
| Q15 | 成为大使对您的职业认同感有何影响? | What impact did become an Ambassador have on your professional identity? | Significantly enhanced; Somewhat enhanced; No effect (Single-choice) |
| **Part 4: Challenges, Needs, and Evaluation** | | | |
| Q16 | 演示刷牙方法时，您最常用的工具是? | What is your most commonly used tool when teaching brushing techniques? | Dental model; Live demonstration (on a person); Video/animation; Verbal explanation only (Single-choice) |
| Q17 | 您更倾向于通过哪些平台发布科普内容? | Which platforms do you prefer for publishing health promotion content? | WeChat/Weibo; Douyin/Kuaishou; Bilibili; Xiaohongshu; Offline bulletin boards; I do not publish content (Multiple-choice) |
| Q18 | 您认为面向受众教学时，哪项口腔保健技能效果最难量化？ | Which oral hygiene skill is the most difficult to quantify in terms of its teaching effectiveness? | Scientific brushing; Dental floss usage; Interdental brush usage; Mouthwash usage; Other (Single-choice) |
| Q19 | 您在口腔科普中遇到的主要困难是? | What are the main difficulties in oral health promotion? (Followed by a 5-point Likert scale on severity for each selected difficulty) | Insufficient time; Lack of high-quality materials; Low acceptance of basic skills (e.g., brushing/flossing) by patients; Insufficient policy support; Other (Multiple-choice) |
| Q20 | 您在开展口腔科普时，是否遇到以下资源不足的情况? | Do you encounter shortages of the following resources when conducting health promotion? | Lack of professional materials (videos, templates); Lack of customized content for different populations (children, elderly); Insufficient demonstration tools (models, props) (Multiple-choice) |
| Q21 | 您认为当前的口腔科普政策或机构支持是否足够？ | Do you think the current policies or institutional support for oral health promotion are sufficient? | Very sufficient, with clear support policies; Average, some support but not systematic; Insufficient, lacking policy guidance and resource allocation; I am completely unaware of related policies (Single-choice) |
| Q22 | 在科普过程中，您的受众对口腔健康知识的接受度如何？ | How would you describe your audience’s receptiveness to oral health knowledge? | Very high, willing to learn and change habits; Average, some are cooperative; Low, most lack interest or have poor compliance; Extremely low, very difficult to promote behavioral change (Single-choice) |
| Q23 | 您认为受众在口腔健康认知上最大的误区是? | What are the biggest misconceptions about oral health among the public? | No pain means no problem, neglecting regular check-ups; Brushing is enough, flossing is unnecessary; Believing myths like dental scaling harms teeth; Other (Multiple-choice) |
| Q24 | 在教授科学刷牙时，您遇到的主要困难是? | What are the main difficulties when teaching scientific brushing methods (e.g., the Bass technique)? | Audience struggles to master the correct technique (angle, pressure); Lack of intuitive demonstration tools (models, animations); Audience believes their current method is fine and is unwilling to change; Other (Multiple-choice) |
| Q25 | 在推广牙线使用时，最大的阻碍是什么? | What are the main difficulties when teaching the use of dental floss? | Audience finds it troublesome; Audience gives up due to gum bleeding; Audience is confused by the variety of floss products on the market; Other (Multiple-choice) |
| Q26 | 在指导牙间隙刷使用时，您认为最难的点是? | What are the main difficulties when teaching the use of interdental brushes? | Audience is unsure if they need to use them; Difficulty in selecting the right size of brush head; Complex operating technique, leading to incorrect use; Other (Multiple-choice) |
| Q27 | 在回答大众关于漱口水的相关问题是，您被问到最多的问题是哪方面? | When answering public questions about mouthwash, what are the most frequently asked questions about? | Ingredients of the mouthwash; Effectiveness in maintaining oral health; Risks of accidental swallowing; Other (Multiple-choice) |
| Q28 | 您认为开展口腔科普的最大时间成本在于？ | What is the largest time cost in conducting oral health promotion? | Preparing content (e.g., creating presentations, videos); The actual execution (e.g., lectures, one-on-one guidance); Follow-up and evaluation (e.g., patient recalls, assessing effectiveness); Other (Single-choice) |
| Q29 | 您如何评估科普效果? | How do you evaluate the effectiveness of your health promotion efforts? | Improvement in patients’ oral health status at follow-up visits; Audience provides feedback on behavioral changes (e.g., starting to use floss); Assessing knowledge retention via questionnaires or tests; Currently lack effective evaluation methods; Other (Multiple-choice) |
| Q30 | 您最需要的科普辅助工具是? | What auxiliary tools for health promotion do you need the most? | Standardized instructional videos (for brushing/flossing); Graphic templates (posters, brochures); A database of frequently asked questions (FAQs) from the public; Other (Multiple-choice) |
| Q31 | 您在口腔健康科普工作中希望获得哪些激励? | What incentives would you like to receive for your work in oral health promotion? | Continuing education credits; Official certificates; Media publicity; Material rewards; Positive feedback from patients; Other (Multiple-choice) |
| *Notes:* All items were presented in a non-randomized order and were included in a survey in Chinese. The survey was conducted in June 2025. | | | |
